# The Role of Vitamin D for Human Health: The Challenge of the Right Study Designs and Interpretation

**DOI:** 10.3390/nu15132897

**Published:** 2023-06-27

**Authors:** Hermann Brenner

**Affiliations:** 1 Division of Clinical Epidemiology and Aging Research, German Cancer Research Center (DKFZ), 69120 Heidelberg, Germany; h.brenner@dkfz.de; 2 Division of Preventive Oncology, German Cancer Research Center (DKFZ), National Center for Tumor Diseases (NCT), 69120 Heidelberg, Germany; 3 German Cancer Consortium (DKTK), German Cancer Research Center (DKFZ), 69120 Heidelberg, Germany; 4 Network Aging Research, Heidelberg University, 69120 Heidelberg, Germany

Numerous observational and intervention studies have suggested adverse health effects of poor vitamin D status and health benefits of vitamin D intake. However, results have often been inconsistent. Plausible explanations for such inconsistencies include heterogeneity in consideration of dose–response relationships in observational studies and the target populations and dosing schemes in intervention studies.

For example, many observational studies found the association between vitamin D status, which is commonly determined by 25-hydroxy-vitamin D (25(OH)D) concentrations in peripheral blood, and various adverse health outcomes, such as total mortality or mortality from various causes, to be close to "L-shaped”. The risk of those outcomes was strongly increased at the lower end of 25(OH)D concentrations, mainly among people with vitamin D deficiency, which is commonly defined by 25(OH)D concentrations below 30 nmol/L. In contrast, the dose–response relationship was essentially flat among people with sufficient vitamin D levels (25(OH)D concentrations > 50 nmol/L). Consequently, very different results can be found and have been obtained for the same associations depending on the distribution of vitamin D status among the population studied, such as strong inverse associations in study populations with a high prevalence of vitamin D deficiency or null associations in study populations with (mostly) sufficient vitamin D status.

Somewhat surprisingly, these very marked dose–response patterns have received rather limited attention in the design of intervention studies to assess the potential health benefits of vitamin D supplementation. For example, in two of the three by far largest such trials, the VITAL study conducted in the US and the D-Health study conducted in Australia, each including more than 20,000 participants, in which 25(OH)D concentration was measured at baseline in subsamples of the participants, mean and median serum 25(OH)D concentrations were above 75 nmol/L. Slightly more than 10% of participants had 25(OH)D concentrations < 50 nmol/L [1,2]. Therefore, it is not surprising that these trials which evaluated the supplementation of vitamin D for people who are unlikely to need it and who are unlikely to benefit from it yielded mostly negative results, i.e., could not demonstrate the benefits of such supplementation [1,3,4,5]. Unfortunately, these findings are widely misunderstood as evidence against the beneficial health effects of vitamin D supplementation. This is particularly concerning since vitamin D insufficiency and deficiency are widespread globally and much more prevalent in most countries than in the US and Australia, where these trials were conducted. An additional concern is that in many studies, including the Australian D-Health study, vitamin D was supplemented in an unphysiological manner, i.e., by rare application of very large bolus doses (e.g., 60,000 International Units once per month in the D-Health study) which most likely is much less efficient or may even be harmful when compared to daily supplementation [6]. 

The goal of this Special Issue, “Dose–Response Relationships of Vitamin D Status and Vitamin D Intake with Health Outcomes”, is to contribute to a better understanding of the health effects of vitamin D status and vitamin D intake by paying adequate attention to specific dose–response relationships of vitamin D status and vitamin D supplementation with a variety of health outcomes, including incidence and mortality of major acute and chronic diseases. Thorough understanding and consideration of such dose–response relationships is a prerequisite for the appropriate design and interpretation of intervention trials aimed at preventing adverse health outcomes by vitamin D supplementation [7].

*Efficacy and safety of personalized vitamin D supplementation*

Previous vitamin D supplementation trials applied the same, uniform doses of vitamin D in the intervention groups, despite high heterogeneity in baseline vitamin D levels within populations. In the German VICTORIA trial, a personalized vitamin D_3_ loading dose (20,000 International Units (IU) per day spread over up to 11 days), followed by a daily maintenance dose of 2000 IU is provided to patients with vitamin D insufficiency several weeks after colorectal cancer surgery [8,9]. In an interim analysis of the first 74 recruited participants, reported in this Special Issue [9], mean 25(OH)D concentrations increased from 25.9 nmol/L at the screening to 63.1 nmol/L after the loading dose phase and 75.5 nmol/L after 12 weeks (end of the maintenance dose phase), and 100% of patients had 25(OH)D concentrations > 50 nmol/L after 12 weeks in the intervention group, compared to only 17.4% in the placebo control group. In none of the patients, 25(OH)D concentrations > 150 nmol/L or hypercalcemia was observed. Rare hypercalciuria events (n = 5 in the verum group and 1 in the placebo group, *p* = 0.209) receded after discontinuing the study medication. This study demonstrated the effectiveness and safety of such a personalized vitamin D supplementation regimen. 

*Relevance of total, bioavailable, or free 25(OH)D*

Approximately 85–90% of total 25(OH)D is bound to vitamin D-binding protein, 10–15% is loosely bound to albumin, and less than 1% is circulating in free form. It has been suggested that bioavailable 25(OH)D (i.e., albumin-bound or free 25(OH)D) or free 25(OH)D alone may better reflect vitamin D status and its associations with health outcomes than a total 25(OH)D. However, a systematic review of studies that evaluated the various biomarkers in parallel, published in this Special Issue, found similar inverse associations of total, bioavailable, and free 25(OH)D with mortality [10]. This finding was confirmed in a recent analysis of the so far largest cohort study assessing these associations [11], which also reported high correlations between the various vitamin D biomarkers [12]. In another analysis from the same large cohort published in this Special Issue, associations with the incidence of diabetes were even stronger for total 25(OH)D than for bioavailable and free 25(OH)D [13]. However, all three biomarkers showed consistent inverse associations with the risk of diabetes among participants with low baseline HbA1_c_ concentrations. Taken together, these results do not support suggestions of switching from a total 25(OH)D to bioavailable or free 25(OH)D when assessing vitamin D status-related health outcomes.

*Vitamin D among specific patient groups*

Although multiple epidemiological studies have found quite consistent evidence of an inverse dose–response relationship between 25(OH)D concentrations and mortality in the general population [14,15] and among specific patient cohorts, such as patients with cancer [16], diabetes [17], hypertension [18], metabolic syndrome [19], and cardiovascular disease [20], such evidence has been sparse for patients with nonalcoholic fatty liver disease (NAFLD). In a study published in this Special Issue, strong inverse dose–response relationships between 25(OH)D concentrations and mortality were found in a large cohort of NAFLD patients identified among participants of the US National Health and Nutrition Survey 2002–2016 [21]. Confirming previously reported results for other general population and patient cohorts, the inverse relationships were very strong among participants with 25(OH)D concentrations below 50 nmol/L (i.e., the range of vitamin D insufficiency and deficiency). In contrast, no such dose–response patterns were seen among participants with sufficient vitamin D status.

A cross-sectional study among more than 15,000 visitors to a health promotion center of an education hospital in Korea, published in this Special Issue, found an inverse dose–response relationship between 25(OH)D concentrations and elevated intraocular pressure, the only major modifiable risk factor for glaucoma [22]. These findings suggest a potential role of vitamin D also for preventing this leading cause of irreversible vision impairment worldwide. Again, the dose–response relationship was much more pronounced in the vitamin D insufficiency and deficiency range than among participants with sufficient vitamin D status. 

## Conclusions

Studies in this Special Issue, along with studies recently reported elsewhere, do not support the suggestion of more informative predictive value for various health outcomes of bioavailable and free 25(OH)D compared to total 25(OH)D. Nevertheless, bioavailable and free 25(OH)D, which are highly correlated with total 25(OH)D, show similarly strong inverse associations with various health outcomes.

Studies in this Special Issue furthermore make important contributions to the accumulating evidence of strong inverse associations of 25(OH)D concentrations with adverse health outcomes within specific patient populations. Of note, in agreement with previous evidence from many other populations and a wide range of other adverse health outcomes, these associations were much stronger among or even restricted to participants with vitamin D deficiency or insufficiency. These results further strengthen suggestions that vitamin D supplementation might be particularly or even exclusively beneficial for people with these conditions. 

Unfortunately, evidence on the health effects of vitamin supplementation from randomized trials is based, to a very large extent, on studies conducted in populations with mostly sufficient vitamin D status. Lack of confirmation of beneficial effects of vitamin D supplementation in such trials may therefore reflect a failure to target the population-at-risk rather than lack of the potential of vitamin D supplementation in preventing premature morbidity and mortality. Other issues that require careful attention in interpreting the intervention trials is the mode of supplementation, given accumulating evidence that regular supplementation of physiological doses rather than occasional very high dose bolus application may be needed to achieve beneficial health effects [23]. Of note, a most recent meta-analysis of 80 randomized trials reported a significant reduction in all-cause mortality by 5% despite including large negative trials conducted in vitamin-D-sufficient populations or applying bolus supplementation [24]. Along with the consistent results from a wealth of observational studies, these patterns suggest an even much larger potential for adequately targeted and applied vitamin D supplementation in preventing morbidity and mortality. Further research is needed to enhance insights in dose–response patterns of vitamin D status and vitamin D supplementation with various health outcomes to fully explore and exploit its preventive potential.
